# Molecular Mechanisms of Tumor Pathogenesis

**DOI:** 10.3390/cells15161452

**Published:** 2026-08-13

**Authors:** Alessandro Lavoro, Saverio Candido

**Affiliations:** 1 Department of Biomedical and Biotechnological Sciences, University of Catania, 95123 Catania, Italy; 2 Research Center for Prevention, Diagnosis and Treatment of Cancer, University of Catania, 95123 Catania, Italy

## 1. Introduction to the Special Issue

Cancer development is a highly complex and dynamic process driven by molecular alterations affecting cellular identity, homeostasis, and interactions with the surrounding environment. In particular, tumor initiation and progression are influenced by a broad spectrum of genetic, epigenetic, and environmental factors that collectively contribute to malignant transformation, cancer cell invasion, and metastasis [1]. Genetic alterations, including somatic and germline mutations, represent key events in tumor pathogenesis by promoting oncogene activation, silencing of tumor suppressor genes, and dysregulation of signaling pathways involved in the regulation of biological processes [2,3]. Similarly, epigenetic mechanisms, including DNA methylation, histone modifications, and non-coding RNA regulation, play a critical role in modulating expression patterns contributing to tumorigenesis. Increasing evidence also highlights the relevance of epigenetic mechanisms in metabolic reprogramming, tumor microenvironment remodeling, and immune regulation [4,5,6]. Given the reversible nature of such alterations, targeting of epigenetic mechanisms has attracted growing interest for the development of novel therapeutic strategies. In particular, CRISPR-Cas-based epigenome editing has recently emerged as a promising approach for precision cancer therapy by modulating cancer-related genes to restore physiological transcriptional patterns without altering the underlying DNA sequence [7,8]. In this context, recent advancements in molecular biology technologies and high-throughput approaches have significantly improved comprehension of the molecular drivers underlying tumorigenesis and cancer progression, revealing novel biomarkers and potential therapeutic targets [9,10,11]. However, the molecular complexity and heterogeneity of tumors continue to represent major challenges, suggesting the need for further investigation aimed to provide a deeper understanding of molecular mechanisms involved in the regulation of cancer. On these bases, the aim of this Special Issue, Molecular Mechanisms of Tumor Pathogenesis, was to provide an updated overview of the molecular mechanisms underlying tumor development, highlighting recent discoveries in tumor biology, emerging therapeutic strategies, and potential cancer biomarkers.

## 2. Overview of Published Articles

The published contributions have investigated tumor complexity by addressing a broad range of biological processes, including post-transcriptional regulation, tumor microenvironment reprogramming, cellular plasticity, epigenetic alterations, and immune regulation. This Special Issue comprises twelve articles, including seven original research articles and five reviews. Among original articles, two studies focused on post-transcriptional regulation in papillary thyroid carcinoma (PTC) development. In particular, Henry et al. explored the role of microRNA-1179 (miR-1179), demonstrating its anticancer activity through the inhibition of cancer cell migration and induction of apoptosis. Mechanistically, miR-1179 expression levels were inversely correlated with *E74 Like ETS Transcription Factor 3* (*ELF3*), *Notch Receptor 3* (*NOTCH3*), and *C-X3-C Motif Chemokine Ligand 1* (*CX3CL1*), suggesting that miR-1179 may counteract PTC progression by simultaneously silencing distinct oncogenic pathways [12]. Rojo-Pardillo et al. also focused on Dicer1, a key ribonuclease involved in microRNA maturation. Using a Cre-Lox system to selectively inactivate *Dicer1* in RET/PTC3 transgenic mice, the authors observed that only the homozygous *Dicer1* loss significantly impaired tumor growth while increasing DNA damage and cancer cell death [13]. Taken together, these studies further confirm the involvement of post-transcriptional regulation in PTC progression, suggesting that these regulatory mechanisms may represent potential therapeutic targets for the development of effective treatment strategies. Another original research article by Ivanova et al. investigated the molecular determinants of colorectal cancer (CRC), evaluating the clinical relevance of YKL-40 and YKL-39 chitinase-like proteins (CLPs) as potential cancer biomarkers. The authors reported opposite expression patterns for these CLPs, with increased plasma YKL-40 and decreased YKL-39 levels in CRC patients compared to controls, indicating distinct roles for these proteins in tumor development, invasiveness, and patient stratification [14]. Liang et al. identified the Hypoxia-Inducible Factor 1 alpha–Prolyl hydroxylase domain-containing protein 1–Forkhead box protein A1 (HIF1α-PHD1-FOXA1) signaling axis as a critical driver of hypoxia adaptation and androgen signaling suppression in prostate cancer. Under hypoxic conditions, HIF1α directly interacted with FOXA1, reducing its protein levels through PHD1-mediated hydroxylation. Targeting this pathway reduced prostate cancer cell proliferation and migration, confirming the therapeutic relevance of this regulatory network in advanced prostate cancer [15].

Beyond molecular alterations driving tumor initiation and progression, cancer cells undergo dynamic adaptive changes that promote aggressive phenotypes, cellular plasticity, and resistance to therapy. In this context, two complementary studies published in this Special Issue investigated molecular determinants of aggressiveness and chemotherapy resistance in urothelial carcinoma (UC). In the first study, the authors demonstrated that *SRY-box transcription factor 2* (*SOX2*) expression was negatively correlated with overall survival and disease-free survival. Interestingly, in vitro *SOX2* knockdown significantly attenuated inflammatory signaling pathways, basal keratin expression, and tumor growth, while increasing sensitivity to cisplatin treatment [16]. The same research group also investigated the involvement of basal keratins (KRTs) in squamous differentiation of UC. Using an in vitro model of UC derived from As3^+^-transformed cells, they observed that *KRT6* silencing was inversely correlated with protein levels of epidermal growth factor receptor (EGFR) and aldehyde dehydrogenase 3 family member A1 (ALDH3A1), which play a key role in cancer stem cell metabolism. Moreover, *KRT6* knockdown reduced cancer cell growth and colony formation, while enhancing cisplatin sensitivity [17]. Taken together, these findings provide novel insights into the role of *SOX2* and *KRT6* in modulating key molecular processes involved in muscle-invasive UC, suggesting that targeting these pathways may represent a promising strategy to overcome chemotherapy resistance and counteract UC progression. Regarding tumor immunity and epigenetic regulation, Leone et al. explored the immune landscape of Triple-Negative Breast Cancer (TNBC) and its relationship with immunotherapy efficacy. Focusing on immune-related genes, the authors identified *Poliovirus Receptor-like 3* (*PVRL3*) as a key prognostic factor in TCGA TNBC patients, whose expression levels were negatively correlated with overall survival. The authors also reported that *PVRL3* was epigenetically regulated by Enhancer of Zeste Homolog 2 (EZH2), as demonstrated by GSK343 treatment and *EZH2* knockdown of cancer cell lines [18]. These results highlight the relevance of epigenetic mechanisms in shaping tumor–immune interactions, suggesting that the EZH2-PVRL3 axis may represent a potential target for personalized therapeutic strategies.

The review articles included in this Special Issue have provided a broader perspective on molecular mechanisms involved in tumorigenesis and discussed emerging therapeutic opportunities. Among these, Dorward et al. reviewed the role of the Tumor Protein D52 (TPD52)-like family in cancer biology, focusing on its involvement in intracellular trafficking, lipid biogenesis, cell proliferation, and cell-cycle regulation. By discussing isoform-specific and tissue-specific tumorigenic roles of TPD52-like family members, the authors highlighted the relevance of these proteins as potential regulators of oncogenic processes [19]. Pzywara et al. provided a systematic review of current therapeutic strategies for mucinous ovarian carcinoma (MOC), a rare subtype of ovarian cancer. The authors also discussed emerging approaches, including antibody–drug conjugates, synthetic lethality approaches, and Wnt/β-catenin pathway inhibitors, highlighting challenges related to tumor heterogeneity and treatment resistance [20]. Rakoczy et al. reported a narrative review on the role of *Ras-associated C3 botulinum toxin substrate 2* (*RAC2*) and *Pituitary tumor-transforming gene 1 product* (*PTTG1*) in the regulation of neoplastic processes, including epithelial–mesenchymal transition, apoptosis inhibition, metastasis promotion, invasiveness, and treatment response, supporting their clinical relevance as cancer biomarkers and therapeutic targets [21]. Another narrative review by Mokhosoev et al. focused on the role of oxidoreductase enzymes belonging to the Cytochromes P450 (CYPs) family in catalyzing the oxidation of several chemicals and the biological effects on anticancer drug susceptibility. By discussing the influence of CYP polymorphism on drug metabolism and anticancer therapy efficacy, particularly in hormone-dependent cancers and hepatocarcinoma, the authors emphasized the need for further investigation to better clarify the role of CYP-mediated mechanisms in tumorigenesis and therapeutic resistance [22]. Finally, Lin et al. provided a comprehensive overview on the potential application of antioxidant-based therapies in cancer treatment. The authors discussed both anticancer activity of antioxidant compounds and issues related to dose-dependent toxicity, redox imbalance, and therapeutic efficacy, highlighting the need for a deeper understanding of oxidative stress regulation in cancer [23].

## 3. Conclusions and Future Perspectives

The articles published in this Special Issue provide novel insights into the molecular mechanisms underlying tumor pathogenesis, highlighting the complexity of cancer biology. In particular, these results demonstrate that tumor pathogenesis and therapeutic response are influenced by several molecular processes, including genetic and epigenetic alterations, transcriptional and metabolic reprogramming, hypoxia, intracellular trafficking, and tumor–immune interactions. Collectively, the biomarkers and therapeutic targets identified in these studies represent a valuable starting point for translational research aimed at elucidating tumor biology and improving patient management. The integration of multi-omics approaches with functional validation and clinical studies will enable a deeper characterization of tumor heterogeneity and promote the translation of molecular findings into personalized treatment strategies, ultimately improving outcomes for cancer patients.
